# On Some Forms of Baldness

**Published:** 1906-11-10

**Authors:** Morgan Dockrell

**Affiliations:** Physician to St. John's Hospital for Diseases of the Skin, Leicester Square


### The Chesterfield Lecture.

### ON SOME FORMS OF BALDNESS.

By Dr. MORGAN DOCKRELL, M.D., Physician to St. John's Hospital for Diseases of the Skin, Leicester Square.

We recently had an opportunity of inspecting this newly-constructed hospital, and have to congratulate the authorities on a building for treating skin diseases which will be found to be in point of equipment and arrangement second to none in Europe. The hospital is finely situated in the centre of a densely-populated neighbourhood, and is provided with all the most recent appliances for the comfort of the patients and of the medical officers who devote their time and skill to treating the patients who come there. The reception-rooms for patients, which are on the ground floor, afford a convenient shelter to those sick persons who formerly had to linger about the square. The consulting-rooms are adequately furnished, fully equipped with dressing-cabinets for the patients and with every diagnostic requirement for the physician. This hospital is entirely devoted to the work of the out-patient department and to the instruction of practitioners who desire further to increase their knowledge of this important branch of their profession. It is provided with the most modern equipment in the shape of $x$-rays, Finsen rays, and Sabouraud's special apparatus for the treatment of ringworm. It is also provided with a magnificent Board-room and secretary's office, and with a completely-equipped laboratory for the investigation of skin diseases and for research purposes. In this hall lectures are periodically given to practitioners by Dr. Morgan Dockrell, Chesterfield Lecturer, and we have much pleasure in reproducing in our pages the first lecture of the present session.

Lecture.

Gentlemen,—Seventeen years ago I had the privilege of giving my first lecture here, and the subject I then took was the same one I have selected this evening. My critics at that time accused me of lecturing while still in the cradle of dermatological knowledge. I

trust I am now in the vigour of at least adult knowledge, and that the lecture given then, composed as it was of the opinions of others to the exclusion of my own, will not now be deemed an impertinence made up as it is of personal observations to the exclusion of ideas put forward so plentifully by other dermatologists.

### Then and Now.

At that time dermatology was more or less in an experimental stage, overburdened with long names, weighted with useless divisions, expounded with but few exceptions by teachers who, not understanding what they taught, were hardly likely to bring conviction to those who listened to them.

Now all this is changed, and here at least we are striving after a simpler dermatology; useless divisions and meaningless terms are being swept away, clinical diagnosis is verified by microscopical section, and dermatology is every day becoming a more accurate science, thus enabling the different diseases to be treated more rapidly, because while formerly treatment was empirical, it is now based on the knowledge of what has taken place, and therefore of that requiring correction.

Passing then more immediately to my subject, let me remind you that hair is not given merely for adornment, but provided for use; and so Nature, while harmonising everything for beauty, giving us coarse hair in different parts of our bodies for protective purposes, provides us in other parts with finer hairs where the needs are not so great, and where civilisation has stepped in to help us. Hair, however, plays an important part in appearance, and the want of it, especially in the male, who is unable to subsidise it as easily as in the other sex, is becoming more an anxiety than formerly. No longer does the young professional man think it necessary to be bald to gain the confidence of his clients, or the city man to impress those with whom he comes in contact with of his great experience. We are all striving to look as young as we can, and to feel as young as we look; and therefore when we find the natural glossiness departing from the hair we use brilliantine to heighten it, and when it begins to come out we fly from one highly-advertised lotion to another, influenced, perhaps, by the portrait of a good-looking journalist with luxuriant hair, who believes it is due to the nostrum he has used all his life and not to his excellent health; or, on the other hand, to the exhibition of wonderful heads of hair which have never even tried the application of the "cure-all" they are called on to sell. Let me remind you, then, that the loss of hair and the want of glossiness are evidences in the early stages of deterioration of health; and when they remain permanent even after health is improved require careful treatment to restore to their normal condition.

Further, I want you to understand that I am not dealing with baldness from ringworm, and that I do not recognise any form of baldness as being caused by a "bacillus" beneath the surface of the skin; nor do I recognise "circular" bald patches as indicative of any particular form of baldness, but capable of occurring in any of the conditions which produce baldness, and therefore limited by some individual circumstance or idiosyncrasy which is not always apparent at the moment.

Baldness is any condition of thinning of the hair of the head which impresses on the observer the appearance of the skin underneath. It may be partial or complete, the latter from gradual extension of disease or suddenly from great shock, as in the case of a young healthy lad brought to me some years ago, who was thrown from a swing, and although at the time not in the least hurt, left all his hair on his pillow on getting up the next morning.

This brings me to the different forms of baldness which I wish you to recognise as due to (1) glandular disturbance, (2) a bacillus, (3) inflammation, (4) a poison in the system, (5) atrophy.

These varieties may give rise to thinning of the hair, but are capable of going on to complete baldness.

### Glandular Disturbance (Seborrhœic Alopecia).

In this form we have two varieties of conditions of the sebaceous glands, due to (1) excessive secretion, (2) diminution of secretion.

Excessive Secretion.—Clinical symptoms.—Here there is great greasiness of the hair, the formation of crusts of yellowish character which are adherent to the scalp, general matting of the hair, which comes out easily, and presents a lank appearance.

*Microscopical Appearances.*—Hair follicles swollen, filled with sebaceous materials, flask bacilli are present, glands at first hypertrophic, later on becoming atrophic.

*Treatment.*—In treating this condition it is well to advise your patients to be careful of tea, coffee, whisky, and excessive use of tobacco.

Internally: Arsenic is beneficial.

Locally: Avoid all greasy applications, and order some lotion of a stimulating character to be used at night, as lysol, 5 per cent., in water or

| | |
|---|---|
| Quiniæ sulph. ... ... ... ... | grs. xvj. |
| Ac. Sulph. dil. ... ... ... ... | ʒij. |
| Tr. Canthar ... ... ... ... | ʒij. |
| Tr. Capsici ... ... ... ... | ʒss. |
| Tr. Lavand co. ... ... ... ... | ʒij. |
| Sp. Vini Rectificat. ad ... ... ... | ℥iv. |
| Ft. lot. | |

Diminution of Secretion.—Clinical symptoms. The hair in this condition presents great dryness, all lustre is gone from it, fine white scales are scattered over the scalp, and there is a general sense of itchiness, the hair coming out freely.

*Microscopical Appearances.*—On section, hair follicle is blocked with horny layer, and there is general atrophy of sebaceous glands.

*Treatment.*—In this variety butter and cream should be partaken of freely.

Internally: Cod-liver oil is useful.

Locally: Antiseptic ointment slightly mercurialised are useful.

| | |
|---|---|
| Hydrarg Ammon. ... ... ... | gr.v. |
| Liq. Carb. Deterg (Wright) ... ... | ♏. xx. |
| Vaselini opt. Flav. ... ... ... | ℥ij. |
| M. | |

To be applied at night.

### 2. Bacilliary Alopecia.

This disease has two stages, one known as trichorrhexis simplex, the other as trichorrhexis nodosa. The condition is due to a gas bacillus, fermentation taking place in the shaft of the hair, and giving rise to certain symptoms.

*Clinical Symptoms.*—A general thinning of the hair takes place, accompanied by a great tendency to either break or split. On taking a hair out and looking along it the contour is irregular, and on pulling it between the fingers the unevenness is easily felt, especially in the nodosa variety.

*Microscopical Appearances.*—At the point of fracture a number of micro-bacilli are to be seen, and the end of hair is brush-like and split often some distance down.

*Treatment.*—Wash with hydronaphthol soap (5 per cent.) once in ten days, and apply an oil with 1 per cent. of hydronaphthol in it, or use the following application:—

| | | |
|---|---|---|
| ℞ | Resorcin ... ... ... ... ... | grs. x. |
| | Ol. Ricini ... ... ... ... ... | ʒss. |
| | Balsam Peruv. ... ... ... ... | ♏ij. |
| | Spt. Vini. Rectif. ad ... ... ... | ℥j. |

### 3. Inflammatory Alopecia (Ul-erythema).

*Clinical Symptoms.*—A patch of redness occurs, more or less circumscribed. This is followed by a loss of hair, and, if inflammation remains unchecked, permanent baldness supervenes, with a scar surrounded by red spreading edges.

*Microscopical Appearances.*—Hair follicle is blocked up with hyaline horny layer; there are hyaline epithelial cells, well-marked mitosis, dilated blood-vessels, infiltration of round cells round the vessels.

*Treatment.*—Internally: Alkalis should be given.

Locally: Treatment must be soothing from the first, as the application of calamine lotion or the positive pole of a continuous current.

### 4. Syphilitic Alopecia.

Here one has a condition very commonly overlooked, although the indications are very remarkable.

*Clinical Symptoms.*—The following symptoms should arouse suspicion: Sudden arrest in growth of some hairs, while in the immediate locality the growth continues, giving the appearance of great irregularity in length. Again the complete loss of hair on one side, presenting an irregular patch. The loss of the arch of the eyebrow on one side. These illustrations all help you to realise better than words can explain.

*Microscopical Appearances.* — Well-marked round-celled infiltration in the cutis surrounding the hair, blocking of blood-vessels, hyaline changes in the cells, and marked peri folliculitis.

*Treatment.*—Internal: One of the different methods that will come under your notice later on when I deal with syphilis generally.

Locally: If there is any evidence of a previous seborrhœa, one of the applications mentioned under this variety should be used.

### 5. Atrophic Alopecia (Alopecia Areata).

In this variety is found a large number of cases which are undoubtedly greatly influenced by deterioration of health, so that the hair is shed locally or universally producing either a limited area of baldness, or involving the whole scalp.

*Clinical Symptoms.*—In the early stages of this condition there is a general wasting of the individual hairs, which fall out and give the appearance of "going bald," or, in more complete cases, well-marked areas of baldness sharply divided off from each other by bands of hair, the scalp surface being smooth, shiny, and shrunken. Often small brush-like stumps are present; these are not, however peculiar to this form of baldness. Later on the whole surface may become bald.

*Microscopical Appearances.* — In the section before you there is atrophy of arrector-pili, of sebaceous gland of hair-shaft and hair-root and capillary vessels. Thrombosis of vessels and marked infiltration of round cells. Micro-bacilli are also found in hair follicles, but these are of no importance as to cause, being found in sections from perfectly healthy scalps and also in sections taken from well-covered scalps after death.

*Treatment.* — Internally: Must be based on general health of patients.

Locally: It must be stimulating. The following formulæ have many advocates:—

| | | |
|---|---|---|
| ℞ | Ol. Sinapis ... ... ... ... ... | ʒj. |
| | Ol. Ricini ... ... ... ... ... | ʒij. |
| | Spt. Rosmarini ... ... ... ... | ʒij. |
| | M. | |

or

| | |
|---|---|
| Liq. Ammon. Fort., Chloroform | |
| Ol. Sesami ... ... ... ... | ʒss. |
| Ol. Limon. ... ... ... ... .. | ʒss. |
| Spt. Rosmarini ... ... ... ad | ℥ij. |
| M. | |

To be rubbed in at first once a day, and then afterwards twice a day:—

| | |
|---|---|
| Ol. Terebinth. Ol. Olivæ... ... aa | ʒij. |
| Tr. Nucis Vomicæ or Tr. Canth. ... | ʒj. |
| Ol. Olivæ ... ... ... ... ... ... | ℥j. |

In conclusion, I would draw your attention to the hatless brigade. The members of this cult go about hatless to encourage the doing of everything from a natural point of view. It is all very well to belong to the hatless brigade so long as you can wander through a large park free from dust, without much exposure to sun, and with good air; but along the country roads full of dust and exposed to the sun I think you will find that the hatless brigade will suffer probably more than they will gain by it.

After each lecture we adjourn to the out-patient department downstairs and take the patients and consider them as they come; and then any question will be answered, so that any of you who may attend these lectures will have an opportunity of getting acquainted clinically with these cases, and in that way be able to grasp the importance of the study of dermatology—one of the few divisions in medicine in which specialists are really necessary.

